# Real-Time Strategy Game Training: Emergence of a Cognitive Flexibility Trait

**DOI:** 10.1371/journal.pone.0070350

**Published:** 2013-08-07

**Authors:** Brian D. Glass, W. Todd Maddox, Bradley C. Love

**Affiliations:** 1 Biological and Experimental Psychology, School of Biological and Chemical Sciences, Queen Mary, University of London, London, United Kingdom; 2 Department of Psychology, University of Texas at Austin, Austin, Texas, United States of America; 3 Institute for Neuroscience, University of Texas at Austin, Austin, Texas, United States of America; 4 Department of Cognitive, Perceptual and Brain Sciences, University College London, London, United Kingdom; Katholieke Universiteit Leuveņ Belgium

## Abstract

Training in action video games can increase the speed of perceptual processing. However, it is unknown whether video-game training can lead to broad-based changes in higher-level competencies such as cognitive flexibility, a core and neurally distributed component of cognition. To determine whether video gaming can enhance cognitive flexibility and, if so, why these changes occur, the current study compares two versions of a real-time strategy (RTS) game. Using a meta-analytic Bayes factor approach, we found that the gaming condition that emphasized maintenance and rapid switching between multiple information and action sources led to a large increase in cognitive flexibility as measured by a wide array of non-video gaming tasks. Theoretically, the results suggest that the distributed brain networks supporting cognitive flexibility can be tuned by engrossing video game experience that stresses maintenance and rapid manipulation of multiple information sources. Practically, these results suggest avenues for increasing cognitive function.

## Introduction

Neuroplasticity is the ability of the adult brain to not only learn new behaviors and form new memories but to alter the underlying neural structures responsible for such learning [1]. This capability can allow for effective compensation against cognitive impairments that coincide with systemic neural changes such as aging or the onset of schizophrenia [2], [3]. One question is whether activities novel to the modern lifestyle can lead to changes in core cognitive functioning. Video gaming represents an immersive and oftentimes intensive activity that is unique to modern humans and is rapidly increasing in popularity. Given the ubiquity of video gaming in modern culture, one important question is whether video games can shape core components of human cognition.

In 2008, 72% of the general population and 97% of teenagers aged 12–17 reported playing video games [4]. Video games are being played more frequently and in more locations: 50% of all teens reported playing “yesterday”, and 60% of all teens play video games on portable devices [5]. Many studies have focused on cognitive differences of video game players, as a group, in comparison to non-gamers [6]–[9]. Although observational studies are essential for studying individual differences, it is also important to examine video game training itself, and the potential causal influences of video gaming on novice players' cognition.

Prior experimental investigation of the cognitive consequences of video gaming provides evidence that cognitive and perceptual changes occur in those who transition from non-gamers to gamers. Specifically, training on action games (e.g., first-person, fast paced, kill-or-be-killed situations) has been linked to enhanced core perceptual processing [10], [11]. Action video game novices assigned to action video game training experience a number of benefits, including higher contrast sensitivity [12], faster visual information processing [13], expanded useful field of view [14], and logical comprehension [15].

One key question is whether video game play can alter aspects of higher-level cognition [16]. In other words, is video game training limited to promoting “fast perception” or can it also promote “fast thinking”? Current findings are promising, but not conclusive. Jaeggi, Buschkuehl, Jonides, and Perrig [17] demonstrated that extended training (8 to 19 days) on an *n*-back test led to higher scores of fluid intelligence (Gf) relative to controls. This is evidence that certain forms of specific training can lead to generalized improvements in higher-level cognition as measured by a test which was entirely different than the training task. In a study of video game training in older adults, Basak, Boot, Voss, and Kramer [18], found that 23.5 hours of training on real-time strategy game led to enhanced executive control and visuospatial skills relative to a non-playing control group. In training studies involving action games, Green et al. [19] and Strobach, French, and Schubert [20] demonstrated a causal link between video game play and improvement in certain executive and cognitive tasks. Oei and Patterson [21] find positive transfer between the skills emphasized by certain types of games and performance in closely related laboratory tasks.

This array of studies is a promising indication that video game training can lead to improvements in cognitive test performance. However, another video game training study carried out on younger adults with little gaming experience did not find improved performance on cognitive testing after 23.5 video game training on either a real-time strategy game, an action video game, or a puzzle game [22]. Overall, previous work indicates video game play can lead to improvements in both perceptual and cognitive performance. The majority of prior research involves action gaming and or games that closely mirror testing conditions. In contrast, we seek to affect broad-based changes in higher-level cognitive ability by emphasizing certain cognitive operations within a single (non-action) video game.

In the present study, we add to an emerging body of literature suggesting video gaming can alter higher-level competencies by devising a gaming intervention and specifying diagnostic measures that are optimized to assess the effects of gaming on cognitive flexibility. Cognitive flexibility is the essential ability to assess and adapt ongoing psychological operations and to coordinate the allocation of cognitive processes appropriately in dynamic decision making environments [23]. Cognitive flexibility is a core aspect of cognition that involves the coordination of cognitive resources in both lower-level perceptual switching and higher-level rule switching [24], and has been associated with fluid intelligence [25] and overall psychological well-being [26]. Cognitive flexibility is likely not subserved by a single neural area, but rather is representative of a broad functional network involving the prefrontal cortex and right superior parietal cortex [27]. One possibility is that broad-based training that engages multiple processes related to cognitive flexibility is required to tune this distributed network. Certain types of video game experience, as opposed to narrow training on a laboratory task, may be well suited for inducing plasticity in the neural systems supporting cognitive flexibility.

Real-time strategy (RTS) game training is an excellent candidate for tuning these cortical networks due to sustained maintenance and rapid switching across multiple information sources at a high workload for long periods of time over several weeks. Our main prediction is that our RTS gaming manipulation will complement previous work in action video games by promoting “fast thinking”, while not strongly affecting “fast perception.” To assess whether video game training can alter cognitive flexibility, the current study utilizes an RTS game, StarCraft (published by Blizzard Entertainment, Inc. in 1998). RTS gaming involves the creation, organization, and command of an army against an enemy army in a real-time map-based setting (see Text S1 for more information on the game). To be successful, the player must cope with simultaneous and rapidly evolving game situations and sub-situations occurring in real-time while managing funds, resources, and information regarding the opponent. Furthermore, we altered the stock StarCraft game program by disabling mini-map alerts, requiring the player to rely on memory for events occurring outside the screen window. In short, while previous work relied on action video games which highlight “fast perception”, RTS gaming highlights “fast thinking” and has been used previously as a successful training regimen. Thus, we predict RTS game training to enhance cognitive flexibility in a general manner.

To further elucidate the role of video gaming on cognitive flexibility, gaming is examined with a within-game and between-game comparison. A life-simulator game is used as a control gaming condition against two versions of the RTS game: a full-map version and a half-map version. The full-map version (SC-2) involves two friendly bases and two enemy bases, whereas the half-map version (SC-1) involves one friendly and one enemy base and half the available gaming space. In the SC-2 version, the player is commanding and controlling two separate bases in multiple battles against two separate opponent bases. For this reason, the SC-2 subcondition promotes more switching and coordination of cognitive resources, hallmarks of cognitive flexibility. Both versions were also modified with a reactive difficulty level in order to maintain a win rate near 50%. Thus, the SC-1 version was designed to be as engrossing and difficult as the SC-2 version, but did not emphasize maintaining awareness of and switching between two spatially separated (out of view) bases. Importantly, game feature and behavior recording within the RTS game allowed for verification of the amount of attended information between the two versions. The life-simulator video game, The Sims 2 (published by Electronic Arts, Inc. in 2004), has been shown to be a useful control for experimental video game research [12]. Together, this is an effort to delineate not only the behavioral characteristics that change in the course of initial video game training, but also the characteristics of the games themselves that lead to change.

To determine changes in cognitive flexibility that occurred as a result of video gaming, participants completed a battery of psychological tasks at pre-test and post-test (at 40 hours of gaming). The battery included measures that address cognitive flexibility as well as measures of unrelated constructs. Measures of flexibility included the Attention Network Test (ANT) [28], Stroop task [29], task switching [30], a novel multi-location memory task, and test of Operating Span (Ospan; distinct from simple counting memory span) [31], [32]. These are classic measures of cognitive flexibility in that they require the switching or coordination of cognitive processes in order to successfully navigate the task at hand. For example, the task switching paradigm involves switching between two different stimulus identification tasks. All the measures in the cognitive flexibility task group assess the ability to coordinate attentional processes between two or more concurrent or alternating operations. Measures of predicted unrelated constructs included the balloon analogue risk task (BART) [33], visual search task [14], information filtering task [34], and WAIS-IV digit span memory task [35], [36]. These tasks were chosen to help delineate the specific hypothesis that RTS training would lead specifically to cognitive flexibility enhancements given that RTS game play stresses fast-paced switching and coordination of decisional processing. The visual search task and the information filtering task were chosen due to their use in previous action video game research [12], in order to differentiate RTS training from action video game training. BART and the digit span memory task were chosen due to further delineate cognitive flexibility from the broad domains of risk sensitivity and general memory. Participant groups were equated on the Multimedia Multitasking Index (MMI), a measure of the amount of time an individual spends simultaneously engaged in more than one form of media [37]. Consistent with best scientific practices and openness, the task grouping and analysis strategy were determined and publically disclosed [38] prior to data collection.

## Methods

**Figure 1:**
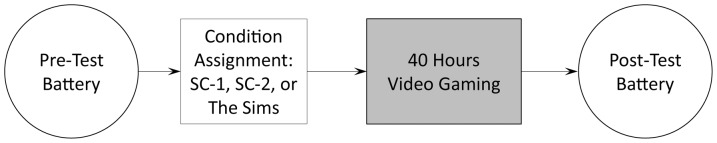
General experimental procedure outline. The experimental procedure included the completion of a pre-test task battery, followed by gaming condition assignment (SC-1, SC-2, or The Sims). Each participant engaged in 40 total hours of video gaming. This video game play occurred outside the lab on the participants' own laptops. Psychological testing occurred over sessions in the laboratory.

Undergraduate participants were recruited from the University of Texas at Austin. Respondents to an advertisement were screened and selected for inclusion on the basis of reported video game use. Participants were randomly assigned to one of the three experimental conditions. Those who reported <2 hours per week of video game use were included (SC-1, n = 26; SC-2, n = 20; Sims, n = 26). Mean age was 20.3 years (SD = 1.1) for SC-1, 20.4 years (SD = 1.1) for SC-2, and 19.9 years (SD = 0.8) for the Sims condition. All participants were female due to the small number of non-gaming males (see Text S1). Primary cognitive testing occurred before video game assignment (pre-test) and after 40 hours of gaming (post-test). Figure 1 illustrates the experimental time-course for all three groups (SC-1, SC-2, and The Sims). Testing occurred over a two day span in a laboratory setting (Table S1). The cognitive battery included standard methodology for all tasks involved. For the multi-location memory task, methodology is available in Text S1. For further information regarding alterations made to the StarCraft game program and downloadable data sets, visit http://brianglass.net.

The video game software along with experiment running software was installed on each participant's laptop. The software provided a portal which controlled the settings for each gaming session and prevented the participant from playing more than 40 hours before post-test. Players were instructed to play for roughly one hour per day. For the SC subconditions, gaming sessions alternated between two StarCraft map formats that differed by terrain theme and layout. Additionally, a titration procedure raised or lowered the difficulty of the following game in response to whether the previous game was won or lost. Difficulty varied between 15 levels and was defined as the amount of production resources available to the opponent entity in the game. This procedure successfully honed win rate (42.6%, SD = 8.8% for SC-1; 43.0%, SD = 8.7% for SC-2). For the Sims condition, participants controlled and developed a single “family household” in a virtual neighborhood. Participants were given 7 weeks (49 days) to complete the study, with the average completion occurring after 43.7 days (SD = 6.24). Further information on materials and methods is available in the Text S1.

### Ethics Statement

All participants gave informed written consent to participate in this study. Approved by the Institutional Review Board of the University of Texas at Austin.

## Results

### Behavioral Modeling of Assessed Cognitive Flexibility

To compare across the various cognitive tasks, and account for the effects of re-test learning, we contrasted post-test enhancements in the RTS subconditions against the control life-simulator condition using diffusion modeling when appropriate, and applying a meta-analytical Bayesian technique [39], [40]. The technique involves a critical second step that is often ignored in null hypothesis significance testing, namely the direct comparison of the likelihood of the null hypothesis to the likelihood of the alternative hypothesis. The Bayes factor technique performs this type of ratio test, resulting in a numeric value representing the likelihood of the alternative hypothesis versus the likelihood of the null hypothesis given the collected data set.

To achieve a meta-analytical Bayes factor approach, we computed a single performance metric for each task. For tasks in which there is accuracy and response time measures, we use diffusion modeling to combine these two measures into a single measure of performance. Diffusion modeling is an established modeling technique for tasks which permit a speed/accuracy tradeoff [41], [42]. For tasks such as the BART, for which diffusion modeling is not possible, we use standardized scoring procedures. Detailed information for each task is available in the Text S1. For each task, performance change was defined as the pre-test to post-test performance difference scores. Importantly, we accounted for repeated exposure learning by comparing both of the RTS subconditions (SC-1 and SC-2) against the Control condition (The Sims 2) performance. In this way, the simple effect of learning from repeated exposure to the tasks was accounted for by making performance change relative to the Control gaming condition. Tables S2, S3, S4, S5, S6, S7, S8, S9, S10 provide detailed task results and subscores.

Using a meta-analytical Bayesian technique that aggregated across multiple *t*-test statistics, we consolidated evidence for or against performance enhancement for the cognitive flexibility tasks and the unrelated tasks. For both SC subconditions, task performance was compared against Control gaming performance using a *t*-test. This comparison accounts for re-test learning that may have occurred from pre-test to post-test. The meta-analytical Bayesian framework combines evidence in an appropriate manner to make a collective assessment regarding evidence for or against the null hypothesis that no performance change has occurred relative to the Control group. The conventional interpretation of the Bayes factor [43] deems ratios greater than 1∶1 as evidence for the alternative hypothesis, with ratios greater than 3∶1 signifying substantial evidence, greater than 10∶1 signifying strong evidence, greater than 30∶1 signifying very strong evidence, and greater than 100∶1 signifying decisive evidence.

The *t*-values used to calculate the meta-analytical Bayes factors and associated effect sizes of the individual tests, as well as means and standard error for pre- and post-test scores, are listed in Tables 1 and 2. Due to the low power of any individual test and the danger of false discovery when performing multiple tests, we refrain from drawing conclusions about the individual tasks and focus on the *a priori* determined meta-analytical Bayesian analyses.

**Table 1:** The *t*-values, which were the basis for calculating the meta-analytic Bayes factors, contrasting post-test minus pre-test scores for SC groups versus the control (Sims) group.

Task Group	Task	SC	SC-1	SC-2
Cognitive Flexibility	Stroop	3.06 (0.53)	1.32 (0.31)	2.84 (0.70)
	ANT	2.28 (0.52)	0.86 (0.32)	2.24 (0.65)
	Task Switching	0.92 (0.18)	0.98 (0.31)	0.23 (0.08)
	Multi-Location Switching	0.98 (0.20)	0.69 (0.17)	0.68 (0.19)
	Ospan	0.81 (0.13)	0.56 (0.15)	0.61 (0.12)
Other	BART	−0.56 (−0.08)	−0.39 (−.08)	0.61 (−0.08)
	Visual Search	−0.58 (−0.13)	0.42 (0.09)	−1.29 (−0.29)
	Information Filtering	−1.00 (−0.20)	−0.29 (−0.08)	−1.34 (−0.34)
	Digit span	−1.76 (−0.22)	−2.66 (−0.47)	−0.25 (−0.04)

Effect sizes (Cohen's *d*) in parentheses.

**Table 2:** The means with standard error in parentheses for pre- and post-test dependent measures (used to calculate meta-analytic Bayes factors) for SC and control (Sims) groups.

Task	Session*	Control	SC-1	SC-2
Stroop	Pre	0.905 (0.183)	0.871 (0.164)	0.667 (0.119)
	Post	0.943 (0.193)	1.106 (0.190)	1.229 (0.169)
ANT	pre	0.019 (0.002)	0.017 (0.002)	0.015 (0.002)
	post	0.024 (0.002)	0.025 (0.002)	0.024 (0.002)
Task Switching	pre	0.011 (0.001)	0.011 (0.001)	0.011 (0.001)
	post	0.013 (0.001)	0.013 (0.001)	0.012 (0.001)
Multi-Location Switching	pre	0.006 (0.000)	0.006 (0.001)	0.006 (0.001)
	post	0.006 (0.001)	0.007 (0.001)	0.007 (0.001)
Ospan	pre	51.60 (3.49)	40.65 (3.85)	44.30 (3.90)
	post	55.70 (4.77)	47.35 (4.69)	50.00 (3.96)
BART	pre	28.66 (2.89)	34.98 (2.17)	27.78 (1.94)
	post	33.05 (2.45)	38.52 (3.18)	31.24 (2.03)
Visual Search	pre	0.014 (0.001)	0.014 (0.001)	0.012 (0.001)
	post	0.018 (0.001)	0.019 (0.001)	0.016 (0.001)
Information Filtering	pre	0.009 (0.001)	0.009 (0.001)	0.009 (0.001)
	post	0.011 (0.001)	0.010 (0.001)	0.009 (0.001)
Digit span	pre	0.391 (0.176)	0.183 (0.211)	0.065 (0.154)
	post	0.948 (0.205)	0.367 (0.193)	0.587 (0.189)

* There were no significant pre-test differences for the StarCraft groups combined versus the Sims control group.

All main predictions held. For the cognitive flexibility task grouping, the Bayes factor for RTS game play (StarCraft) versus the control game (The Sims) was 40.76. As predicted, benefits for RTS gaming only held for the cognitive flexibility task grouping. For the other tasks, the Bayes factor for RTS game play (StarCraft) versus the control game (The Sims) was 0.02, which signifies very strong evidence for the null hypothesis. When comparing the SC-1 and SC-2 subconditions directly, no strong evidence for differences was found. However, when considering the cognitive flexibility task grouping, the SC-2 subcondition did differ from the Sims control game (Bayes factor of 6.77), whereas the evidence for SC-1 subcondition differing from the Sims control game was inconsequential (Bayes factor of 1.17). Overall, these results are highly supportive of our predictions – RTS gaming selectively promotes cognitive flexibility, particularly under conditions in which players must rapidly switch between contexts while maintaining memory for both contexts.

### Principal Component Analysis of a Cognitive Flexibility Dimension

One key question is whether an underlying dimension of cognitive flexibility emerges or strengthens as a result of video game experience. A principal component analysis (PCA) on the absolute cognitive task scores (as opposed to post-test minus pre-test difference scores as compared to the Sims control condition) revealed the main underlying performance components within each game group. At pre-test, neither the Sims nor the SC group (SC-1 and SC-2) had a pattern of primary principle components, which aligned with the hypothesized cognitive flexibility task distinction. However, at post-test, the SC group's first principle component organized along the hypothesized cognitive flexibility task distinction. To demonstrate the emergence of this pattern of focused improvement on cognitive flexibility, the vector of PCA component contributions for each task were correlated with the hypothesized contribution factor vector (1 for cognitive flexibility tasks, 0 for unrelated tasks). For pre-test, the correlations for the Sims and SC conditions were not significant, *r* = −0.06, *p* = 0.88 and *r* = 0.14, *p* = 0.73, respectively (with no significant distinction between the two correlations, *z* = 0.35, *p* = 0.73). However, at post-test, the SC group correlated significantly, *r* = 0.94, *p*<0.001, whereas for the Sims condition the correlation with the cognitive flexibility component remained non-significant, *r* = −0.44, *p* = 0.24 (with the distinction between the two correlations statistically significant, *z* = 3.83, *p*<0.001). Thus, after 40 hours of RTS video gaming, those in the SC group exhibited a performance component that is aligned with cognitive flexibility.

Taken together, the PCA and meta-analytical BF results reveal that the RTS video game training paradigm successfully enhanced and aligned the cognitive performance of the group along a new and unified dimension of cognitive flexibility. Forty hours of RTS video game training was sufficient to create dramatic changes in players' cognitive flexibility.

### RTS Game State Feature Analysis

Our previous analyses indicate that those in the SC-2 subcondition, but not necessarily those in the SC-1 subcondition, benefited from enhanced cognitive flexibility as a result of RTS training. We originally hypothesized that RTS gaming involved the practice of maintaining, assessing, and coordinating between multiple information and action sources simultaneously. In order to verify that the SC-2 subcondition stresses these operations more than the SC-1 subcondition, we analyzed players' behavior within the RTS game. In particular, we examined players' use of game features to test the hypotheses that SC-2 players simultaneously process more within game information than SC-1 players. See the Text S1 for further details on the modification of the StarCraft gaming engine and for behavioral data.

During RTS game play, game state features and user behavior were recorded every 250 milliseconds. Game features (e.g., whether a unit is being attacked) are used to predict which unit is selected by the player. By constructing the selection/feature analysis for time step lags into the past, it became possible to determine which features in the near past drove user selection in the present. In this way, we implement Bayesian model selection to determine how many features a player entertains.

**Figure 2:**
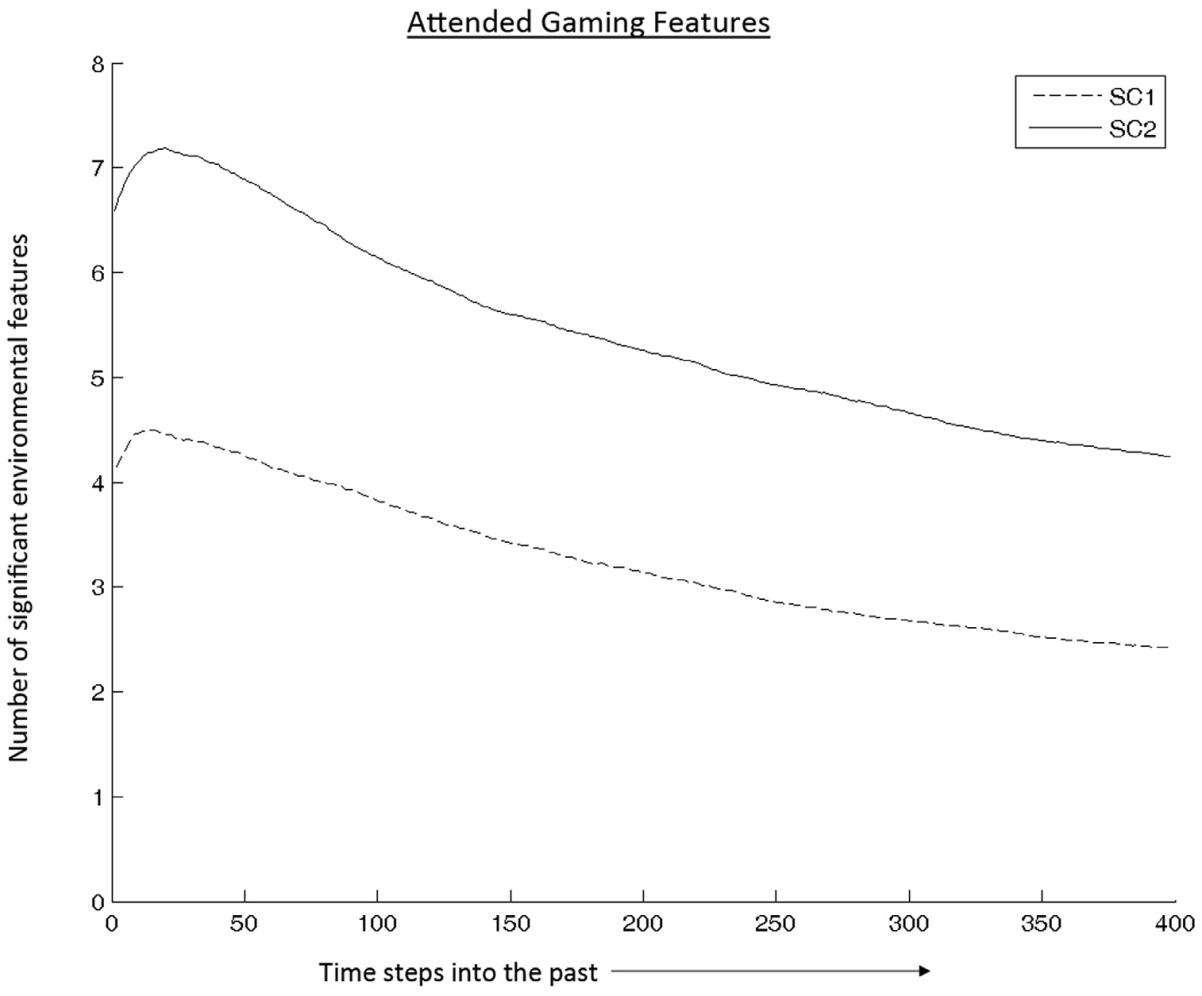
Size of attentional feature set that drives behavior at the current (0 seconds) time step. As time progresses, fewer gaming features from the past influence current game play behavior. The SC-2 group attended to more features and at post-test exhibited enhanced cognitive flexibility performance relative to the Sims group.

Figure 2 illustrates the number of significant game state features for the SC subconditions at various time lags. Those in the SC-2 subcondition utilized more game state features than those in the SC-1 subcondition. This result supports the notion that the within-StarCraft gaming manipulation led SC-2 participants to manage more information sources during game play, thus leading to enhancements in cognitive flexibility.

## Discussion

The present study finds that cognitive flexibility is a trainable skill. Forty hours of training within an RTS game that stresses rapid and simultaneous maintenance, assessment, and coordination between multiple information and action sources was sufficient to affect change. As a result of RTS game experience, an underlying dimension of cognitive flexibility emerged and characterized individual differences in performance on a variety of laboratory tasks.

Secondary analyses involved comparing SC-1 and SC-2 subconditions. Comparing the SC-1 and SC-2 individually to the baseline control game, SC-2, which involved maintaining awareness of and switching between two spatially separated (out of view) bases, was particularly effective in boosting players' cognitive flexibility. By recording real-time gaming data, we were able to compare the two StarCraft groups in terms of the number of significant features attended during game play. This has revealed that in fact the SC-2 gaming setup led to more attended features overall than SC-1.

This delineation of gaming environment and the resulting gaming behavior is a novel demonstration that begins to clarify which gaming attributes are important for meaningful cognitive change. We have shown that training with a sufficient level of simultaneous information and action coordination in a real-time video game leads to the specific enhancement of higher-level cognition along a clear unified component. With the ability to control and quantify specific video game parameters and behavior, we have shown that it is possible to alter cognitive flexibility, a core component with broad influence on the psychological abilities and well-being of an individual. We have also shown that only one version of RTS gaming led to cognitive flexibility enhancements while another did not. The SC-2 subcondition was designed to require even more switching and maintenance of information. Modeling of the gaming behavior indicated that those in the SC-2 subcondition were attending to more gaming features. These aspects of RTS gaming may have been the critical elements of the video gaming training regimen required to elicit a cognitive flexibility enhancement.

One key direction for future research is to detail the brain basis for these dramatic changes in behavior. Promising avenues include structural magnetic resonance imaging (MRI) to explore volumetric changes to brain regions following video game experience, as well as diffusion tensor imaging (DTI) to explore changes in connectivity between brain regions as a result of training. Given the nature of cognitive flexibility, we expect that changes will not be localized to one region. Understanding the brain basis for the behavior change may suggest how to best pair varieties of game experience with populations seeking to improve cognitive flexibility. Applications of this innovation include the development of clinical regimens to target deficits in populations with specific cognitive flexibility or executive functioning dysfunction. These include attention deficit hyperactivity disorder (ADHD) [44], complications with normal aging [45], and traumatic brain injury [46].

## Supporting Information

Text S1 **Supporting methods.**
(DOC)

Figure S1 **Difficulty level reached by map type for SC-2 and SC-1.** Error bars represent standard error. This reflects similar game difficulty and engagement between SC-2 and SC-1.
(TIF)

Table S1 Latin square task counterbalancing (MMI = Multimedia Multitasking Index, BART = Balloon Analog Risk Taking, TS = Task Switching, DS = WAIS-IV Digit Span, ANT = Attention Network Test, Ospan = Operating Span, IF = Information Filtering, VS = Visual Search, MLM = Multi-location Memory).
(DOCX)

Table S2 **Task Switching task, post-test minus pre-test, with standard error in parentheses.**
(DOCX)

Table S3 **Stroop test, post-test minus pre-test, with standard error in parentheses.**
(DOCX)

Table S4 **ANT, post-test minus pre-test, with standard error in parentheses.**
(DOCX)

Table S5 **Multi-location memory task, post-test minus pre-test, with standard error in parentheses.**
(DOCX)

Table S6 **Ospan test, post-test minus pre-test, with standard error in parentheses.**
(DOCX)

Table S7 **Visual search task, post-test minus pre-test, with standard error in parentheses.**
(DOCX)

Table S8 **WAIS-IV digit span test, post-test minus pre-test, with standard error in parentheses.**
(DOCX)

Table S9 **Information filtering task, post-test minus pre-test, with standard error in parentheses.**
(DOCX)

Table S10 **Balloon Analog Risk Taking, post-test minus pre-test, with standard error in parentheses.**
(DOCX)
