# Supplementary material for: Real-Time Strategy Game Training: Emergence of a Cognitive Flexibility Trait
Source: PLoS One. 2013 Aug 7;8(8):e70350. doi: 10.1371/journal.pone.0070350 (PMC3737212; doi:10.1371/journal.pone.0070350)
Supplement: Text S1 — Supporting methods. (DOC) [file pone.0070350.s001.doc]

**Glass, Maddox, and Love - Text S1**

**Materials and Methods**

This section provides methodological details, further explanation of the task battery analysis, and details regarding the StarCraft game feature modeling. Methodological details provided here include procedural information, participant information, and explanation of the task battery. For further information regarding alterations made to the StarCraft game program and downloadable data sets, visit [http://brianglass.net](http://brianglass.net/).

*Overall Experimental Procedure*

In this section, the procedures are detailed. Additionally, the section includes the justification for which tasks are incorporated into the Cognitive Flexibility task group or the Other task group. Figure 3 illustrates the experimental time course for all three groups (SC-1, SC-2, and The Sims). Participants were given 7 weeks (49 days) to complete the study, with the average completion occurring after 43.7 days (SD = 6.24). The task battery included the Cognitive Flexibility test group (Stroop task, Attention Network Test, Task Switching, Multi-location Memory Task, and Operating Span) and the Other test group (Balloon Analog Risk Taking test, Visual Search, Information Filtering, and WAIS-IV Digit Span). Task ordering was counterbalanced using a Latin square (Table S1), with the task ordering maintained on each of the three testing sessions. For simplicity, the current analysis focuses on the first and last testing sessions, and the second testing session is available as a downloadable data set from the online depository. The Multimedia Multitasking Index (MMI) questionnaire was given on pre-test (1st session) only. In total, ten different orderings were used.

*Participants*

Potential participants were screened with a web-based questionnaire regarding their current and past video gaming habits. The principle items of the questionnaire were, “In the last year, how many hours per week do you tend to play video games?” and “Prior to the last year, think back to the period in which you most frequently played video games. How many hours per week did you tend to play during that period?” Participants who reported 2 hours or less of video game play per week qualified for inclusion in the study. A sample of 816 prospective participants (463 female) were screened for inclusion in the present study. Only nine male respondents qualified based on their self-reported gaming habits, whereas 90 females qualified. For this reason, we utilized female participants. For all male respondents, the median hours of current weekly game play was 8 (SD = 9.20) and 20 hours (SD = 18.8) during their most frequent period of gaming. For all female respondents, the median hours of current weekly game play was 1 (SD = 7.08) and 7 (SD = 12.6) at their most frequent. Participants were also asked to indicate the genres of video games they currently play, and no included participants endorsed real-time strategy games.

Qualified participants were randomly assigned to one of the three conditions: SC-2 (n = 20), SC-1 (n = 26), or Sims (n = 26). The mean age was 20.4 years (SD = 1.1) for the SC-2 condition, 20.3 years (SD = 1.1) for the SC-1 condition, and 19.9 years (SD = 0.8) for the Sims condition. Average hours of self-reported current weekly game play was 0.38 (SD = 0.55). All participants had normal or corrected to normal vision, and none were colorblind. The groups did not significantly differ in their MMI scores for multimedia multitasking (4.31, SD = 1.64 for SC-1; 4.71, SD = 1.57 for SC-2; 4.40, SD = 1.51 for Sims), and were similar to a standardized general population mean of 4.38 (SD = 1.52) reported by the index’s creators. The Institutional Review Board of the University of Texas at Austin approved the study and informed consent was obtained from all participants.

*Video Game Installation and Tutorials*

After completion of the pre-test, each participant brought their personal laptop to the experimenter for video game installation. The Windows operating system was installed on a drive partition if the participant’s computer was an Apple. To streamline the video game running procedure, we developed a special computer program to automatically run the video game program with a single click. This program also recorded the cumulative time of each gaming session, and did not allow the participant to exceed the gaming hour limit.

After game installation, the experimenter described the video game and instructed the participant to complete tutorials before beginning video gaming. Existing tutorials from the game developers were used, as they taught the essentials of game play. Participants in the StarCraft conditions were additionally provided with a game information packet which described game play in further detail. All participants were informed that our special computer program was recording their gaming and keeping track of their time.

For StarCraft, the computer program recorded hundreds of features from each gaming unit (like complex chess pieces) every 250msec. These features included information such as whether the unit was near friendly or enemy units, current proportion of health remaining, time since last under attack, etc. Importantly, the computer program also recorded which gaming units the participant was currently selecting. This allows for future analysis of in-game performance. For The Sims, saved game information was recorded which carries information about the characters and neighborhood built by the participant during game play.

*Video Gaming Procedure*

For StarCraft, we altered the stock StarCraft game program by disabling mini-map alerts, requiring the player to rely on memory for events occurring outside the screen window. Each gaming session consisted of a single match versus a computer opponent. Two game maps were used and the computer program automatically set up the game so that the maps alternated between gaming sessions. Game lengths ranged between 10 minutes and 80 minutes. Alternating maps allows for varied game play to keep the participant engaged across sessions, while also offering a cross-validation paradigm for future data analysis projects that will analyze the feature and selection information of the gaming units. In addition to the variation in game maps, a titration procedure was developed to adapt the difficulty of the computer opponent to the success of the participant. Depending on if the participant won or lost the previous game, the following game would either be one level more or less difficult, respectively. Fifteen levels existed for each map, with higher levels involving more difficult computer opponents (represented by offering the computer opponent more starting resources). The highest level attained was 13 by one participant, but see below for more details.

For The Sims, each gaming session consisted of monitoring and controlling a single virtual household in a virtual neighborhood. Each household consisted of various family members, as characters (called Sims) would often die or be born. The participant controlled various domestic activities such as buying furniture, finding a job, planting a garden, and interacting with neighbors. All participants were instructed to not play more than three hours of video games per day, and to refrain from playing the games in small time increments. To ensure that all participants had completed the same amount of video gaming, the experiment software limited game play to 40 hours.

**Results Methodology**

*Psychological Task Battery Analysis*

To measure performance changes in the StarCraft conditions, it was necessary to 1) account for re-test effects, and 2) contrast against the control Sims condition. To accomplish this, we utilized a meta-analytical framework to measure the effect of StarCraft game play on Cognitive Flexibility tasks and Other tasks. First, speeded choice tasks were analyzed using a diffusion modeling approach, while the remaining tasks were analyzed using standard methods. Second, for all tasks and all conditions, a post-test minus pre-test difference score was computed. Third, for both StarCraft conditions (SC-1 and SC-2), the difference scores for each task were contrasted against the Sims condition difference scores. Therefore, the resulting t-statistics represent performance enhancements from pre-test to post-test relative to the control group. This conservative approach accounts for re-test effects and furthermore ensures that reported performance enhancements are relative to a Control condition. The t-statistics for Cognitive Flexibility tasks are combined using a Bayesian meta-analytical approach to generate a single Bayes factor to represent the overall effect of StarCraft game play relative not only to pre-test but also to the control Sims condition. A Bayes factor is also computed for the Other task group. The Bayesian approach allows for significance testing of both the alternative hypothesis that StarCraft gaming led to cognitive flexibility enhancements relative to control as well as the null hypothesis that StarCraft gaming did not enhance cognitive flexibility relative to control.

*Diffusion Modeling*

Computerized tasks with two-choice response characteristics and for which reaction time and accuracy are available lend themselves to diffusion modeling. Diffusion modeling determines whether performance changes in speeded reaction time tasks represent true cognitive enhancements or simply speed-accuracy tradeoffs. Because it is common in prior research in video game training to draw conclusions about performance in speeded RT tasks, it is important to rigorously examine potential speed-accuracy tradeoffs.

The EZ-diffusion model simulates trials as a scenario in which evidence accumulates over time in the direction of one of two response boundaries. Responses are made when the evidence marker reaches one of the two response bounds. The EZ-diffusion model takes into account the mean and variance of response times for correct responses as well as response accuracy to estimate a drift rate parameter which corresponds to the quality of information utilized in the decision making system.

Two measures were taken to ensure the speeded choice data was appropriate for diffusion modeling. First, reaction time outliers ±2 SD from the mean were removed. Second, accuracies of exactly 1 were specially handled. For accuracies of exactly 1, edge-correction should be applied to convert the accuracy using Equation S1, where n is the number of trials.

$$A_{new}=\frac{1}{2n}$$

*The Sims as Control Baseline*

Repeated administration of cognitive tasks often results in practice effects, which have been shown to occur over retest intervals of minutes, days, and weeks. To combat this issue, The Sims condition is used as a control baseline condition against which SC-1 and SC-2 are compared. Performance change from pre-test to post-test are calculated as a difference score. Comparisons are then made between the SC condition and Sims. In this way, retest effects are subtracted from the SC condition performance metrics.

Below, Tables S2-S10 report various subtest scores and measures from the tasks. Note that it is often difficult to determine a speed-accuracy tradeoff simply by perusing the mean accuracy and median reaction time of a given participant or group of participants. For this exact reason, diffusion modeling provides insights into potential speed-accuracy tradeoffs.

*Task Switching (Cognitive Flexibility Task Group)*

The task switching paradigm is a dual stimulus-classification procedure during which the participant must switch back and forth between two different task modes. During one mode, the participant identifies a letter as a vowel or consonant. During the other mode, the participant identifies a number as even or odd. After training on both modes, the participant switches between the two task modes. Switch costs are calculated by the reaction time difference between items which followed an item from the same task mode versus items which occurred after a switch in task mode.

On each trial, participants were cued for 200msec with "NUMBER" or "LETTER", depending on the trial type. The stimuli consisted of a number/letter pair (e.g., "4n" or "n4"). On number trials, the participant indicated whether the number was even or odd. On letter trials, the participant indicated whether the letter was a vowel or consonant. Letters were chosen from the set {a, e, i, u, p, k, n, s}; Numbers were chosen from the set {2, 4, 6, 8, 3, 5, 7, 9}. The interval between cue and stimulus onset was 226msec, while the inter-trial interval was 950msec. First, the participant completed three practice sets: letter task only, number task only, and switching practice. Next, the participant completed four 80 trial test blocks. Each block had a 40% switch rate, and all contained the same number of 1-, 2-, 3-, and 4-streak sequences. The task takes approximately 20 minutes to complete. Analysis consisted of diffusion modeling with the resulting drift rate parameter as the main dependent variable.

*Stroop Test (Cognitive Flexibility Task Group)*

The Stroop Test involves three verbal subtests during which the experimenter counts the number of correct and error responses within 45 seconds. First, the participant reads a list of words which include “Red”, “Blue” and “Green” in random order and printed in black ink. Second, the participant is presented with a list of X’s printed in red, blue or green ink and must say the color of the X’s aloud. Lastly, the participant is presented with a list of the three color words in which every word is printed in a different color ink than indicated by the printed word itself (e.g., “Red” printed in green ink). For each subtest, the experimenter instructs the participant to read the printed items aloud (or say the color of the printed items) as quickly as possible. The experimenter counts the number of completed items along with the number of errors made by the participant in 45sec. The task takes approximately 4 minutes to complete. The scores from the three subtests are combined using standardized procedures to create an interference score, which is the main dependent variable.

*The Attention Network Test (Cognitive Flexibility Task Group)*

The ANT is a flanker identification task with various trial types that can be contrasted to measure different forms of attention. On any given trial, a fixation cross is presented (pre-fixation phase), followed by a cue, followed by a fixation phase, followed by a target item flanked by two items on the left and another two on the right. The target is always an arrow pointing either left or right, while the flankers are either all arrows (directional flankers) or lines (neutral flankers). The cue is a fixation cross that is sometimes accompanied by an asterisk either at fixation, above, below, or both above and below. Following the cue, the target item and flankers are presented either above the fixation cross or below.

Task timing was such that the pre-fixation phase varies randomly between 400 and 1600ms. The cue remains visible for 100ms and the fixation phase lasts 400ms. Next, the target items are displayed in a response-terminated fashion. Finally, an inter-trial interval lasts for 3500ms minus the pre-fixation phase duration and minus the recorded reaction time for the present trial. The experiment consists of a 24-trial practice block, followed by two experimental blocks of 96 trials.

Three types of cues are used: no cue (fixation with no asterisk), center cue (asterisk at fixation), double cue (asterisks above and below fixation), or spatial cue (asterisk above or below fixation). Three types of flankers are used: congruent flankers (arrows in the same direction as target), incongruent flankers (arrows in the opposite direction as target), or neutral (lines with no directionality). Trial variation in cues and flankers allows for three critical contrasts: alerting (no cue minus double cue trials), orienting (center cue minus spatial cue), and executive control (incongruent flanker minus congruent flanker). Analysis consisted of diffusion modeling with the resulting drift rate parameter as the main dependent variable

*Multi-location Memory Task (Cognitive Flexibility Task Group)*

A novel memory task was developed for the purpose of the present investigation. We found that the above task switching paradigm did not require retention of the state of the previous task when switching. Other researchers have attempted to tackle this problem using a primary task which is interrupted by a secondary task, throughout which the state of the primary task must be retained. Both tasks were the same and involved spelling the word ‘tablet’ one letter at a time. We expanded on this idea by developing a paradigm involving math operations. For this task, one of two screen locations is active for any given trial. After a single initialization trial during which two green zeros are shown at both location, the participant is shown a white number at one of the locations. The participant responds whether the sum of this white number and the previous green number shown at that location is odd or even, and then a new green number is shown at that location. Thus, the participant must retain the number at the previous location in order to do well.

The participant completed a 30-trial practice block followed by a 100-trial testing block. Before the first trial of each block, two green zeros are shown simultaneously for 0.5 seconds, one at the left location and one at the right location. Then, the zeros are removed and the block begins. Throughout the block, each location is indicated by a white rectangle. On each trial, one of the two locations is cued by the white rectangle becoming thicker for 0.5 seconds. Then, a white number is shown at the cued location (picked randomly from 1 to 9). The participant then indicated whether the sum of the current white number and the green number to be previously shown at that location was even or odd. The white number was cleared from the screen upon response. After 250msec, a new green number was shown at the current location for 0.5 seconds. The next trial began after this 0.5sec delay. The location was selected randomly with equal probability on each trial, although the maximum streak length was set to 10. This is exceedingly rare, since a streak of 10 occurs on only 1 out of 210 trials. In the practice block, corrective feedback and detailed instructions were displayed for the first 10 trials to ensure the participant understood the instructions. The entire task tasks approximately 15 minutes to complete. Analysis consisted of diffusion modeling with the resulting drift rate parameter as the main dependent variable.

*Operating Span (Cognitive Flexibility Task Group)*

In the automated Ospan, participants used the mouse to respond to math equations and identify strings of memorized letters. On each trial, a letter is shown on the screen for 800msec. Then, an unsolved math operation is shown. The participant was instructed to click the mouse once she solved the operation. The operation was cleared from the screen and replaced by a number. The participant signaled whether the number was the correct solution to the operation by clicking on buttons labeled "True" or "False". Next, a new letter is shown. After a determined set of letters had been shown, the participant was presented with a matrix of letters and was instructed to recall the previous string of letters. The experiment includes three sets of each string size from 3 to 7, resulting in 75 trials. The Ospan score was calculated using only strings which were successfully recalled without error. The total number of trials from these successful sets was summed into one final score, which is the main dependent variable. The task takes approximately 10 minutes to complete.

*Visual Search (Other Task Group)*

The visual search task involves the identification of a target in an array of items. There are two trial types: hard and easy. Easy trials have fewer items (4), and hard trials have more (12). All items were represented by white bars on 75% gray disks against a 25% gray background. The target item, if present, had a vertical white bar. Non-target items had white bars that were rotated 45 degrees clockwise or counterclockwise. There were an equal number of target and non-target trials, as well as an equal number of easy and hard trials, with the ordering determined randomly. Items were located randomly on the vertices of an imaginary six by six array (36 possible locations) with vertices spaced 100 pixels apart horizontally and vertically. Disks were 40 pixels in diameter; bars were 6 pixels in width. On each trial, a fixation cross was displayed for 500msec, followed by the response terminated item array. Corrective feedback was displayed for 1sec, followed by a 500msec inter-trial interval. The task consisted of two blocks, each containing 5 practice trials and 96 test trials. The task took approximately 15 minutes to complete. Analysis consisted of diffusion modeling with the resulting drift rate parameter as the main dependent variable.

*WAIS-IV Digit Span (Other Task Group)*

The WAIS-IV digit span task involves remembering and repeating back sequences of numbers ranging in length from 2 to 9, and both forward and backward. For each length, the participant attempts to recall two sequences. If the participant recalls at least one of the sequences correctly, then the experimenter proceeds to the next sequence. This repeats until the participant is unable to recall either sequence. A combined accuracy z-score is calculated using standard procedures. The resulting z-score is the main dependent variable. The task takes approximately 4 minutes to complete.

*Information Filtering (Other Task Group)*

The information filtering task involves the detection of change (or lack of change) in sequentially presented scenes. Participants were shown scenes consisting of red and blue rectangles with differing orientation for 100msec. They were instructed to ignore the blue rectangles and focus only on the red rectangles. The scene was removed for 900msec and then replaced with a new scene. Participants were asked to respond within 2000msec whether one of the red rectangles had been rotated in the new scene. The target red rectangle was either not rotated or rotated by 45 degrees clockwise or counterclockwise. Thus, the participant must filter the scene information by ignoring blue bars and detecting whether red bars have changed. The inter-trial interval was set to 200msec. Scenes included 0, 2, 4 or 6 blue distractors, and 2, 4, 6, or 8 red distractors. The rectangles did not overlap (nor were within one rotation of overlapping) and were distributed evenly across the scene. The total number of rectangles never exceeded 8, thus 10 combinations were possible. There were an equal number of combinations shown, trial order was randomized across subjects, and the number of change and no-change trials was equivalent. There are 256 trials, and the task takes approximately 25 minutes to complete. Analysis consisted of diffusion modeling with the resulting drift rate parameter as the main dependent variable.

*Balloon Analog Risk Taking Task (Other Task Group)*

The Balloon Analog Risk Taking Task (BART) involves decisions regarding when to cash in on monotonically increasing rewards for which the risk of losing the entire reward increases as well. The scenario is depicted graphically with an ever expanding balloon that pops when it reaches a predetermined size drawn from a normal distribution around a mean size (unknown to the participant). The optimal strategy (i.e., the optimal ratio of risk to reward) converges on a pop rate of 50%. Risky strategies will have higher pop rates, while conservative strategies will have lower pop rates.

The experiment consists of 10 balloons, with each balloon ending when either the balloon pops or the participant decides to cash in on the reward. At the beginning of each balloon, the temporary amount of reward collected returns to 0, while the cumulative amount of collected reward remains on the screen. On each trial, the participant chooses to either pump the balloon to cash in on the reward. On the first trial, if the participant chooses to pump the balloon, it will explode with a probability of 1/128. The probability of explosion is 1/127 on the second trial, 1/126 on the third trial, etc. On the 128th trial, the balloon always explodes. Thus, the average breakpoint occurred after 64 pumps. The participant’s average number of pumps over 10 balloons was the main dependent variable. The task took approximately 8 minutes to complete.

*The Multimedia Multitasking Index (Pre-test Only)*

The MMI is an index of simultaneous media consumption. The participant is presented with a matrix of possible media combinations and self reports how often he or she simultaneously engages in both of the media types. These pair-wise reports are weighted with self reported time spent engaging in each medium, and then summed into one score. The developers of the measure found that MMI correlates negatively with the above mentioned information filtering and task switching tasks. In other words, those who tend to multitask more often tend to also be worse at task switching and information filtering.

The questionnaire has two phases: 1) Hours spent on each medium, 2) Pair-wise multitasking frequency. 1) The participant indicated how many hours per week she spends engaging in the following media types: Print media, Television, Computer-based video (e.g., YouTube), Music, Non-music audio, Video or computer games, Telephone and mobile phone voice calls, Instant messaging, Text messaging, Email, Web surfing, and Other computer-based applications (e.g., word processing). 2) A matrix was displayed of each pair-wise combination, and the participant responded for each combination how often she simultaneously engages in both forms of media (e.g., Telephone while Web surfing) -- The options were "Most of the time", "Some of the time", "A little of the time", and "Never". Rows and columns were blacked out for media types which the participant responded 0 hours/week in part one. The questionnaire takes approximately 5 to 10 minutes to complete.

For scoring purposes, the matrix responses were assigned numerical values of 1, 0.67, 0.33, and 0. Then, a sub-score for each media type was calculated by multiplying the matrix response by the number of hours, and dividing this product by the total number of hours. These sub-scores were then summed into one final MMI score, which was the main dependent variable.

*Bayesian Meta-Analysis of Psychological Task Battery*

Our main hypothesis is that a certain form of video game play can lead to the enhancement of a broad and important psychological attribute, namely cognitive flexibility. To test this hypothesis, we employ a host of psychological tests, some designed to gauge cognitive flexibility, and some that gauge alternative constructs. By using a technique from meta-analytical statistics, we are able to test our main hypothesis by considering the evidence provided by a group of psychological tests. We use a Bayesian meta-analytical approach to test the hypothesis that video game play leads to enhanced cognitive flexibility as measured by a series of relevant tests. To further elucidate the claim that any detected enhancement is not of a general psychological nature, we use this same approach on a series of alternative tests that are designed to measure abilities other than cognitive flexibility.

An individual Bayes factor is a ratio between the likelihood function of two models given some set of data. Thus, the Bayes factor can be used as an alternative to classical hypothesis testing in that no Gaussian or other distributions are referenced or assumed. Instead, the ratio of the probability of an alternative model is pitted against the probability of a null model, given some set of data. Unlike likelihood-ratio tests which use maximum likelihood parameter estimation, Bayes factors are calculated by integrating over the parameter space. Equation S2 is the basic form of an individual Bayes factor.

$$BF=\frac{\Pr(D|M_{1})}{\Pr(D|M_{2})}=\frac{\int\Pr(\Theta_{1}|M_{1})\Pr(D|\Theta_{1},M_{1})d\Theta_{1}}{\int\Pr(\Theta_{2}|M_{2})\Pr(D|\Theta_{2},M_{2})d\Theta_{2}}$$

, where *D* represents the empirical data, *A* represents the alternative hypothesis, *N* represents the null hypothesis, and *θ* represents the parameter set. Although one might suspect that multiple Bayes factors can be combined via multiplication, since both the numerator and denominator of the equation represent probabilities, this is not the case. This can be shown by randomly drawing several simulated results from a single underlying distribution. The individual Bayes factors will be small and often lower than 1, thus their product will likely be small despite all experiments being driven by the same underlying distribution. It can be verified that when the smaller data sets are pooled, or concatenated into one larger data set as if combined into one experiment, the resulting Bayes factor is large. However experiments generally cannot (and should not) be pooled due to practical differences. In other words, one should not concatenate two raw data sets if there were methodological differences between the two experiments. Thus, the meta-analytic Bayes factor unifies distinct experimental results via the common language of the t-statistic.

The meta-analytic extension for the Bayes factor has a one-tail and two-tail form, depending on whether a Cauchy or half-Cauchy distribution is used as a prior on effect size. In the present study, we have no expectation given prior research that the Sims condition will outperform the SC-2 and SC-1 groups on post-test, thus we seek to contrast what we hypothesize to be an effect (RTS game training) versus no effect (Sims game training). For this reason, the half-Cauchy distribution is used.

Similar to classical, frequentist approaches to model comparisons, there is no quantitative threshold for determining whether the alternative model is favored over the null. Instead, we rely on an interpretative norm: ratios less than 1:1 support the null hypothesis, 1:1 to 3:1 are insubstantial, 3:1 to 10:1 are substantial, 10:1 to 30:1 are strong, 30:1 to 100:1 are very strong, and ratios greater than 100:1 are decisive.

*Analysis of StarCraft Game Play*

The StarCraft gaming environment was modified such that gaming features and player behavior were recorded every time step. The StarCraft time step is the smallest unit of time in the StarCraft gaming engine, and lasts 250 milliseconds. On each time step, specific characteristics of each gaming unit are recorded. Gaming units are defined as the player’s collection of ground and air combat units as well as buildings. For each unit, a collection of binary and continuous features are recorded (e.g., distance from nearest Command Center, number of visible enemy units nearby, time since last attacked). Critically, the software records whether the unit was currently selected by the player. For the full feature list and more details about the programming modification that makes this possible, visit: [http://brianglass.net](http://brianglass.net/).

*StarCraft Gaming Results*

Two procedural mechanisms were put in place to keep participants engaged in StarCraft gaming and limit within- and between-condition variation in gaming difficulty. First, games alternated between two different gaming maps. Second, a difficulty titration procedure was used. When a participant won a game, the following game featured an opponent that was slightly more difficult to defeat. Likewise, losing a game caused the opponent in the following game to be easier to defeat.

The titration procedure had the expected effect on the proportion of games won. On average, those in the SC-1 condition won 42.6% of their games (SD = 8.8%). Those in the SC-2 condition won 43.0% of their games (SD = 8.7%). This demonstrates that the conditions did not differ by gaming success.

Final game level reached (ranging from 1 to 15) for SC-1 was 4.17 (SD = 2.52) in the Interior-Enemy Map, and 2.50 (SD = 1.25) for Exterior-Enemy Map. Final game level reached for SC-2 was 3.09 (SD = 1.44) in Map 1, and 2.83 (SD = 1.15) in Map 2. Figure S1 illustrates the level reached over time for SC-2 and SC-1.
*Feature Modeling*

The game feature analysis was conducted after all games have been completed by all participants. First, continuous game features were converted into multiple binary features using a binning procedure. For each continuous feature, the full histogram of values is created by considering each game from each participant. Four bin quantiles are constructed by considering the range of the recorded feature values. The continuous feature is then recomputed into a vector of four binary features, representing the bin quartile in which any given feature value falls. In this way, the complete binary feature vector for each game. Second, these binary features for each gaming unit are then compared to whether the gaming unit was selected by the player or not. This comparison is calculated at a range of time steps in the past, from 0 (current time) to 400 time steps (100 seconds) in the past. For each binary feature at each time stop (0 to 400), the four possibilities were 1) Feature On, Selection On, 2) Feature On, Selection Off, 3) Feature Off, Selection On, and 4) Feature Off, Selection Off. Third, these tallied combinations were used in a model comparison analysis to determine whether the gaming features were associated with gaming behavior (unit selection).

The model comparison technique involved the calculation of Bayes factors, or ratios of the likelihood of the empirical data given a feature-aware model versus the likelihood of the empirical data given a feature-blind model. The Bayes factor is calculated for a given feature *f* as in Equation S3,

$$BF_{f}=\frac{\int_{p=0}^{1} p^{k}\left[\left(1-p\right)^{n-k}\right]}{\int_{p_{1}=0}^{1} \int_{p_{2}=0}^{1} \left[p_{1}^{k_{1}}\left(1-p_{1}\right)^{n_{1}-k_{1}}\right]\left[p_{2}^{k_{2}}\left(1-p_{2}\right)^{n_{2}-k_{2}}\right]}$$

, where *k* is the total number of positive selections (selection on), *k_1_* is the number of positive selections when feature is on, k_2_ is the number of positive selections when feature is off, *n* is the total number of selections (selection on or off), *n_1_* is the number of selections when feature is on, and *n_2_* is the number of selections when feature is off.

**Supporting Information Legends:**

Figure S1. Difficulty level reached by map type for SC-2 and SC-1. Error bars represent standard error. This reflects similar game difficulty and engagement between SC-2 and SC-1.

Table S1*.* Latin square task counterbalancing (MMI = Multimedia Multitasking Index, BART = Balloon Analog Risk Taking, TS = Task Switching, DS = WAIS-IV Digit Span, ANT = Attention Network Test, Ospan = Operating Span, IF = Information Filtering, VS = Visual Search, MLM = Multi-location Memory)

Table S2. Task Switching task, post-test minus pre-test, with standard error in parentheses.

Table S3. Stroop test, post-test minus pre-test, with standard error in parentheses.

Table S4. ANT, post-test minus pre-test, with standard error in parentheses.

Table S5. Multi-location memory task, post-test minus pre-test, with standard error in parentheses.

Table S6. Ospan test, post-test minus pre-test, with standard error in parentheses.

Table S7. Visual search task, post-test minus pre-test, with standard error in parentheses.

Table S8. WAIS-IV digit span test, post-test minus pre-test, with standard error in parentheses.

Table S9. Information filtering task, post-test minus pre-test, with standard error in parentheses.

Table S10. Balloon Analog Risk Taking, post-test minus pre-test, with standard error in
