# Supplementary figures and images for: Real-Time Strategy Game Training: Emergence of a Cognitive Flexibility Trait
Source: PLoS One. 2013 Aug 7;8(8):e70350. doi: 10.1371/journal.pone.0070350 (PMC3737212; doi:10.1371/journal.pone.0070350)

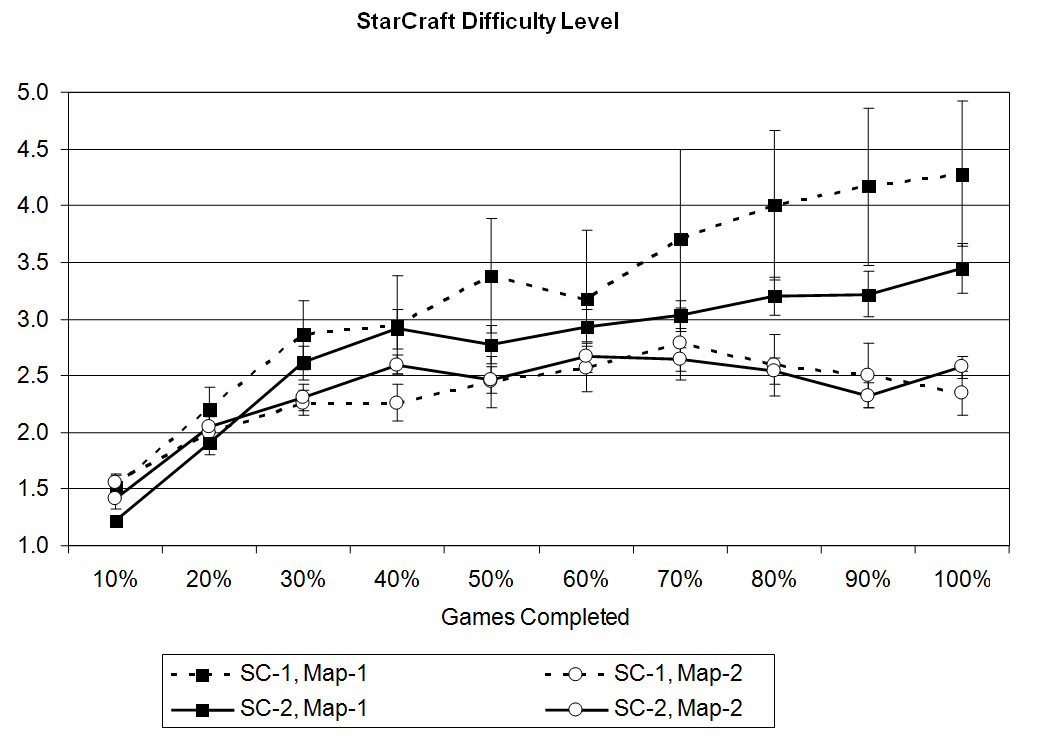

Supplement: Figure S1 — Difficulty level reached by map type for SC-2 and SC-1. Error bars represent standard error. This reflects similar game difficulty and engagement between SC-2 and SC-1. (TIF) [file pone.0070350.s002.tif]
