# Supplementary material for: Real-Time Strategy Game Training: Emergence of a Cognitive Flexibility Trait
Source: PLoS One. 2013 Aug 7;8(8):e70350. doi: 10.1371/journal.pone.0070350 (PMC3737212; doi:10.1371/journal.pone.0070350)
Supplement: Table S1 — Latin square task counterbalancing (MMI = Multimedia Multitasking Index, BART = Balloon Analog Risk Taking, TS = Task Switching, DS = WAIS-IV Digit Span, ANT = Attention Network Test, Ospan = Operating Span, IF = Information Filtering, VS = Visual Search, MLM = Multi-location Memory). (DOCX) [file pone.0070350.s003.docx]

Table S1*.*

| **Task** | **1st** | **2nd** | **3rd** | **4^th^** | **5th** | **6th** | **7th** | **8th** | **9th** | **10th** |
| --- | --- | --- | --- | --- | --- | --- | --- | --- | --- | --- |
| 1 | MMI | MLM | BART | VS | Stroop | IF | TS | Ospan | DS | ANT |
| 2 | BART | MMI | Stroop | MLM | TS | VS | DS | IF | ANT | Ospan |
| 3 | Stroop | BART | TS | MMI | DS | MLM | ANT | VS | Ospan | IF |
| 4 | TS | Stroop | DS | BART | ANT | MMI | Ospan | MLM | IF | VS |
| 5 | DS | TS | ANT | Stroop | Ospan | BART | IF | MMI | VS | MLM |
| 6 | ANT | DS | Ospan | TS | IF | Stroop | VS | BART | MLM | MMI |
| 7 | Ospan | ANT | IF | DS | VS | TS | MLM | Stroop | MMI | BART |
| 8 | IF | Ospan | VS | ANT | MLM | DS | MMI | TS | BART | Stroop |
| 9 | VS | IF | MLM | Ospan | MMI | ANT | BART | DS | Stroop | TS |
| 10 | MLM | VS | MMI | IF | BART | Ospan | Stroop | ANT | TS | DS |
