# Supplementary material for: Real-Time Strategy Game Training: Emergence of a Cognitive Flexibility Trait
Source: PLoS One. 2013 Aug 7;8(8):e70350. doi: 10.1371/journal.pone.0070350 (PMC3737212; doi:10.1371/journal.pone.0070350)
Supplement: Table S2 — Task Switching task, post-test minus pre-test, with standard error in parentheses. (DOCX) [file pone.0070350.s004.docx]

Table S2.

| **Task Switching** | **The Sims** | **SC-1** | **SC-2** | **SC-1 vs Control**  **(*t*-value)** | **SC-2 vs Control**  **(*t*-value)** |
| --- | --- | --- | --- | --- | --- |
| Drift Rate | 0.001 (0.001) | 0.002 (0.001) | 0.001 (0.001) | 0.983 | 0.232 |
| Accuracy | -0.021 (0.016) | 0.015 (0.020) | 0.012 (0.016) | 1.757 | 2.066 |
| Median RT | -172.421 (54.378) | -117.313 (31.575) | -199.594 (54.378) | 1.745 | -0.5 |
| Switch Cost (Accuracy) | -0.008 (0.012) | 0.013 (0.008) | -0.024 (0.012) | 2.732 | -1.277 |
| Switch Cost (RT) | -51.278 (25.602) | 4.652 (51.014) | -51.333 (25.602) | 1.096 | -0.002 |
