# Supplementary material for: Real-Time Strategy Game Training: Emergence of a Cognitive Flexibility Trait
Source: PLoS One. 2013 Aug 7;8(8):e70350. doi: 10.1371/journal.pone.0070350 (PMC3737212; doi:10.1371/journal.pone.0070350)
Supplement: Table S3 — Stroop test, post-test minus pre-test, with standard error in parentheses. (DOCX) [file pone.0070350.s005.docx]

Table S3.

| **Stroop Test** | **The Sims** | **SC-1** | **SC-2** | **SC-1 vs Control**  **(t-value)** | **SC-2 vs Control**  **(t-value)** |
| --- | --- | --- | --- | --- | --- |
| Standard Z-score | 0.038 (0.184) | 0.235 (0.150) | 0.562 (0.184) | 1.317 | 2.84 |
| Accuracy  (Words block) | 0.004 (0.001) | 0.002 (0.002) | 0.002 (0.001) | -0.802 | -1.65 |
| RT (Words block) | -44.618 (7.392) | -41.646 (16.435) | -26.478 (7.392) | 0.181 | 2.454 |
| Accuracy (Color block) | -0.002 (0.003) | 0.010 (0.004) | -0.002 (0.003) | 2.722 | 0.064 |
| RT (Color block) | -54.858 (10.987) | -31.161 (11.158) | -22.362 (10.987) | 2.124 | 2.958 |
| Accuracy (Interference block) | 0.013 (0.007) | 0.011 (0.006) | -0.009 (0.007) | -0.388 | -3.153 |
| RT (Interference block) | -76.448 (20.747) | -81.742 (27.139) | -116.541 (20.747) | -0.195 | -1.932 |
| Accuracy (Overall) | 0.004 (0.002) | 0.007 (0.003) | -0.002 (0.002) | 0.998 | -2.95 |
| RT (Overall) | -55.190 (8.033) | -48.206 (12.685) | -40.337 (8.033) | 0.551 | 1.849 |
