# Supplementary material for: Real-Time Strategy Game Training: Emergence of a Cognitive Flexibility Trait
Source: PLoS One. 2013 Aug 7;8(8):e70350. doi: 10.1371/journal.pone.0070350 (PMC3737212; doi:10.1371/journal.pone.0070350)
Supplement: Table S4 — ANT, post-test minus pre-test, with standard error in parentheses. (DOCX) [file pone.0070350.s006.docx]

Table S4.

| **ANT** | **The Sims** | **SC-1** | **SC-2** | **SC-1 vs Control**  **(t-value)** | **SC-2 vs Control**  **(t-value)** |
| --- | --- | --- | --- | --- | --- |
| Drift Rate | 0.005 (0.002) | 0.008 (0.003) | 0.010 (0.002) | 0.856 | 2.239 |
| Accuracy | 0.011 (0.032) | 0.063 (0.035) | 0.096 (0.032) | 1.51 | 2.649 |
| Median RT | -61.868 (18.813) | -46.136 (24.427) | -80.412 (18.813) | 0.644 | -0.986 |
| Executive Control Score (RT) | -0.011 (0.055) | -0.083 (0.049) | -0.120 (0.055) | -1.457 | -1.985 |
| Executive Control Score (Accuracy) | 0.022 (0.094) | 0.174 (0.105) | 0.283 (0.094) | 1.448 | 2.778 |
| Alerting Score (RT) | 0.038 (0.030) | 0.014 (0.025) | 0.062 (0.030) | -0.957 | 0.804 |
| Alerting Score (Accuracy) | -0.018 (0.015) | -0.034 (0.016) | -0.009 (0.015) | -1.03 | 0.626 |
| Orienting Score (RT) | -0.001 (0.025) | -0.031 (0.021) | -0.047 (0.025) | -1.475 | -1.866 |
| Orienting Score (Accuracy) | -0.008 (0.014) | 0.035 (0.019) | 0.005 (0.014) | 2.236 | 0.918 |
