# Supplementary material for: Real-Time Strategy Game Training: Emergence of a Cognitive Flexibility Trait
Source: PLoS One. 2013 Aug 7;8(8):e70350. doi: 10.1371/journal.pone.0070350 (PMC3737212; doi:10.1371/journal.pone.0070350)
Supplement: Table S5 — Multi-location memory task, post-test minus pre-test, with standard error in parentheses. (DOCX) [file pone.0070350.s007.docx]

Table S5.

| **Multi-Location Memory** | **The Sims** | **SC-1** | **SC-2** | **SC-1 vs Control**  **(t-value)** | **SC-2 vs Control**  **(t-value)** |
| --- | --- | --- | --- | --- | --- |
| Drift Rate | 0.001 (0.001) | 0.001 (0.001) | 0.001 (0.001) | 0.693 | 0.682 |
| Accuracy | -0.020 (0.032) | 0.007 (0.033) | 0.034 (0.032) | 0.802 | 1.714 |
| Accuracy (Switch Trials) | 0.001 (0.053) | 0.046 (0.043) | 0.080 (0.053) | 1.054 | 1.503 |
| Accuracy (Non-switch Trials) | -0.043 (0.015) | -0.036 (0.027) | -0.008 (0.015) | 0.263 | 2.323 |
| Switch Cost (Accuracy) | 0.043 (0.046) | 0.081 (0.027) | 0.088 (0.046) | 1.421 | 0.965 |
| Median RT | -374.613 (109.042) | -227.763 (106.489) | -44.860 (109.042) | 1.379 | 3.024 |
| Median RT (Switch Trials) | -566.025 (160.944) | -239.369 (153.991) | -14.439 (160.944) | 2.121 | 3.427 |
| Median RT (Non-switch Trials) | -318.536 (97.232) | -168.233 (115.468) | -118.759 (97.232) | 1.302 | 2.055 |
| Switch Cost (RT) | -247.489 (122.122) | -71.136 (90.934) | 104.320 (122.122) | 1.939 | 2.881 |
