# Supplementary material for: Real-Time Strategy Game Training: Emergence of a Cognitive Flexibility Trait
Source: PLoS One. 2013 Aug 7;8(8):e70350. doi: 10.1371/journal.pone.0070350 (PMC3737212; doi:10.1371/journal.pone.0070350)
Supplement: Table S6 — Ospan test, post-test minus pre-test, with standard error in parentheses. (DOCX) [file pone.0070350.s008.docx]

Table S6.

| **Operating Span** | **The Sims** | **SC-1** | **SC-2** | **SC-1 vs Control**  **(t-value)** | **SC-2 vs Control**  **(t-value)** |
| --- | --- | --- | --- | --- | --- |
| Standardized Z-Score | 4.100 (2.632) | 6.706 (4.674) | 5.700 (2.632) | 0.558 | 0.608 |
