# Supplementary material for: Real-Time Strategy Game Training: Emergence of a Cognitive Flexibility Trait
Source: PLoS One. 2013 Aug 7;8(8):e70350. doi: 10.1371/journal.pone.0070350 (PMC3737212; doi:10.1371/journal.pone.0070350)
Supplement: Table S7 — Visual search task, post-test minus pre-test, with standard error in parentheses. (DOCX) [file pone.0070350.s009.docx]

Table S7.

| **Visual Search** | **The Sims** | **SC-1** | **SC-2** | **SC-1 vs Control**  **(t-value)** | **SC-2 vs Control**  **(t-value)** |
| --- | --- | --- | --- | --- | --- |
| Drift Rate | 0.004 (0.001) | 0.004 (0.001) | 0.003 (0.001) | 0.422 | -1.285 |
| Accuracy | -0.001 (0.013) | 0.005 (0.008) | -0.006 (0.013) | 0.705 | -0.345 |
| Median RT | -148.368 (30.825) | -129.618 (20.933) | -191.525 (30.825) | 0.896 | -1.4 |
| Distractor Cost (Accuracy) | 0.001 (0.010) | -0.012 (0.010) | 0.004 (0.010) | -1.241 | 0.296 |
| Distractor Cost (RT) | -149.026 (51.174) | -111.588 (31.025) | -159.125 (51.174) | 1.207 | -0.197 |
