# Supplementary material for: Real-Time Strategy Game Training: Emergence of a Cognitive Flexibility Trait
Source: PLoS One. 2013 Aug 7;8(8):e70350. doi: 10.1371/journal.pone.0070350 (PMC3737212; doi:10.1371/journal.pone.0070350)
Supplement: Table S8 — WAIS-IV digit span test, post-test minus pre-test, with standard error in parentheses. (DOCX) [file pone.0070350.s010.docx]

Table S8.

| **WAIS-IV Digit Span** | **The Sims** | **SC-1** | **SC-2** | **SC-1 vs Control**  **(*t*-value)** | **SC-2 vs Control**  **(*t*-value)** |
| --- | --- | --- | --- | --- | --- |
| Standardized *Z*-Score | 0.557 (0.142) | 0.183 (0.146) | 0.522 (0.142) | -2.564 | -0.245 |
