# Supplementary material for: Real-Time Strategy Game Training: Emergence of a Cognitive Flexibility Trait
Source: PLoS One. 2013 Aug 7;8(8):e70350. doi: 10.1371/journal.pone.0070350 (PMC3737212; doi:10.1371/journal.pone.0070350)
Supplement: Table S9 — Information filtering task, post-test minus pre-test, with standard error in parentheses. (DOCX) [file pone.0070350.s011.docx]

Table S9.

| **Information Filtering** | **The Sims** | **SC-1** | **SC-2** | **SC-1 vs Control**  **(t-value)** | **SC-2 vs Control**  **(t-value)** |
| --- | --- | --- | --- | --- | --- |
| Drift Rate | 0.002 (0.001) | 0.001 (0.001) | 0.000 (0.001) | -0.288 | -1.336 |
| Accuracy | 0.020 (0.018) | 0.017 (0.032) | 0.005 (0.018) | -0.085 | -0.794 |
| Median RT | -66.031 (25.955) | -54.972 (21.180) | -63.867 (25.955) | 0.522 | 0.083 |
| Median RT (0 Distractors) | -79.750 (28.638) | -81.111 (33.588) | -71.900 (28.638) | -0.041 | 0.274 |
| Median RT (2 Distractors) | -71.750 (32.133) | -71.444 (45.431) | -50.800 (32.133) | 0.007 | 0.652 |
| Median RT (4 Distractors) | -71.156 (41.416) | -119.306 (27.825) | -45.100 (41.416) | -1.73 | 0.629 |
| Median RT (6 Distractors) | -42.469 (49.604) | 32.611 (51.535) | -92.200 (49.604) | 1.457 | -1.003 |
| Accuracy (0 Distractors) | 0.035 (0.021) | 0.014 (0.033) | 0.014 (0.021) | -0.645 | -1.026 |
| Accuracy (2 Distractors) | 0.033 (0.025) | 0.010 (0.040) | -0.023 (0.025) | -0.581 | -2.261 |
| Accuracy (4 Distractors) | -0.032 (0.029) | 0.053 (0.046) | 0.028 (0.029) | 1.832 | 2.108 |
| Accuracy (6 Distractors) | -0.004 (0.033) | 0.022 (0.037) | 0.049 (0.033) | 0.703 | 1.595 |
