# Supplementary material for: Real-Time Strategy Game Training: Emergence of a Cognitive Flexibility Trait
Source: PLoS One. 2013 Aug 7;8(8):e70350. doi: 10.1371/journal.pone.0070350 (PMC3737212; doi:10.1371/journal.pone.0070350)
Supplement: Table S10 — Balloon Analog Risk Taking, post-test minus pre-test, with standard error in parentheses. (DOCX) [file pone.0070350.s012.docx]

Table S10.

| **BART** | **The Sims** | **SC-1** | **SC-2** | **SC-1 vs Control**  **(t-value)** | **SC-2 vs Control**  **(t-value)** |
| --- | --- | --- | --- | --- | --- |
| Standardized Z-Score | 4.386 (2.347) | 3.535 (2.182) | 3.466 (2.347) | -0.39 | -0.392 |
